# Erratum to: Study on the Greek demographic chart of psychic disorders

**DOI:** 10.1186/s12991-017-0130-x

**Published:** 2017-02-13

**Authors:** Anastasia Karkanis, John Kouros, Demy Kotta

**Affiliations:** Association of Psychology & Psychiatry for Adults & Children, Athens, Greece

## Erratum to: Annals of General Psychiatry 2008, 7(Suppl 1):S327 DOI 10.1186/1744-859X-7-S1-S327

The authors have revised the order of the author list for this article [1]. John Kouros has been moved from 1st to 2nd, Demy Kotta has been moved from 2nd to 3rd and Anastasia Karkanis has been moved from 3rd to 1st.
